# Ketamine and Esketamine in Obsessive–Compulsive Disorder: A Scoping Review of Clinical and Mechanistic Evidence

**DOI:** 10.3390/ph19040628

**Published:** 2026-04-16

**Authors:** Maria Marmureanu, Mariana Valy Besoiu, Vlad Dionisie, Mihnea Costin Manea, Catalin Pleșea-Condratovici, Sorana Iulia Voican, Mirela Manea

**Affiliations:** 1 Department of Adult Psychiatry, “Prof. Dr. Alexandru Obregia” Clinical Hospital of Psychiatry, 041914 Bucharest, Romania; 2 Department of Social Medicine, “Carol Davila” University of Medicine and Pharmacy, 020021 Bucharest, Romania; 3 Titan Psychiatric Hospital “Dr. Constantin Gorgos”, 030447 Bucharest, Romania; 4 Department of Psychiatry and Psychology, “Carol Davila” University of Medicine and Pharmacy, 020021 Bucharest, Romania; 5 Morphological and Functional Sciences Department, Faculty of Medicine and Pharmacy, “Dunărea de Jos” University of Galați, 800008 Galati, Romania

**Keywords:** obsessive–compulsive disorder, OCD, ketamine, esketamine

## Abstract

**Background/Objective**: A substantial proportion of patients with obsessive–compulsive disorder (OCD) does not respond adequately to first-line treatments such as selective serotonin reuptake inhibitors and cognitive-behavioral therapy. OCD has traditionally been conceptualized as a serotonergic disorder. However, emerging evidence suggests that glutamatergic dysfunction plays an important role. Ketamine and esketamine are NMDA receptor antagonists with rapid antidepressant effects and have therefore attracted interest as potential treatments for OCD. This scoping review aims to map and synthesize the existing preclinical and clinical evidence regarding the therapeutic potential of ketamine and esketamine in OCD. **Methods**: A scoping review methodology based on the Arksey and O’Malley framework and Joanna Briggs Institute guidance was applied. Searches were conducted in PubMed, Scopus, and Web of Science. Studies that examined ketamine or esketamine in OCD populations or relevant experimental models were included. **Results**: Twenty-one studies met the inclusion criteria, of which five were preclinical studies and sixteen were clinical investigations. Preclinical evidence suggests that ketamine and esketamine improve compulsive-like behaviors. Clinical studies suggest that ketamine can produce rapid reductions in obsessive symptoms, though results remain inconsistent. Most trials evaluated single administrations, while limited evidence suggests that repeated dosing strategies may provide greater clinical benefit. **Conclusions**: Ketamine and esketamine show promise as rapid acting interventions for OCD, particularly in treatment refractory cases. However, current evidence remains preliminary and heterogeneous. Future research should prioritize adequately powered randomized trials and investigation of repeated administration protocols with longer follow-up periods to determine efficacy and optimal clinical implementation.

## 1. Introduction

A substantial proportion of patients with OCD fail to respond adequately to standard treatments such as SSRIs and CBT. Augmentation strategies are often necessary to improve clinical outcomes and quality of life [1]; however, commonly used approaches, such as SSRI augmentation with atypical antipsychotics, have shown only modest and clinically limited efficacy [1]. Approximately one-third of treatment-refractory patients achieve meaningful symptom reduction following antipsychotic augmentation. Its clinical utility is further constrained by tolerability issues, such as metabolic and extrapyramidal side effects [2,3]. CBT-ERP and SSRIs often require weeks to months to achieve meaningful symptom reduction [4]. All these limitations, including delayed onset of action, partial treatment response and tolerability issues, underscore the ongoing need for augmentation strategies that are both mechanistically distinct and more robust than currently available options.

Traditional models of OCD have emphasized serotonergic dysfunction [5]. However, increasing evidence suggests the role of glutamatergic abnormalities [6]. These models are interconnected, with serotonergic systems exerting modulatory effects on glutamatergic neurotransmission, particularly within the CSTC circuits. These comprise interconnected loops linking the orbitofrontal cortex, anterior cingulate cortex, striatum, and thalamus, and are critically involved in action selection, inhibitory control, and habit formation. More specifically, the CSTC circuitry is organized into parallel, partially segregated functional loops (limbic, associative, and motor), each contributing differently to OCD symptom dimensions. The limbic loop, involving the orbitofrontal cortex and ventral striatum, is particularly implicated in the generation of intrusive thoughts and affective dysregulation. The associative loop, linking the dorsolateral prefrontal cortex with the dorsal caudate, contributes to impaired cognitive flexibility and maladaptive decision-making [7].

The abnormal glutamatergic transmission within this circuit leads to a dysregulated thalamic input and impaired cortical inhibition. This dysfunction is further characterized by hyperactivity within orbitofrontal–striatal pathways and impaired feedback regulation. Such alterations contribute to persistent intrusive thoughts and compulsive behaviors [8]. OCD has been associated with an imbalance between the facilitatory and inhibitory pathways of the basal ganglia, which leads to excessive thalamic excitation of cortical regions [9]. This imbalance is thought to be driven, in part, by glutamatergic hyperactivity and reduced GABAergic inhibitory control within the striatum [10].

This interplay may provide a potential explanation for both the partial efficacy of SSRIs and the emerging therapeutic relevance of glutamatergic agents. The strongest support comes from cerebrospinal fluid studies that prove elevated glutamate levels in unmedicated patients with OCD, complemented by genetic and neuroimaging findings of abnormal glutamate signaling [11,12,13].

Neuroimaging studies that used resting-state functional MRI and diffusion tensor imaging have demonstrated increased connectivity between the orbitofrontal cortex and striatum, alongside reduced top-down control from prefrontal regions [14,15].

Considering these findings, glutamatergic modulation has emerged as a promising therapeutic approach [16]. A range of glutamatergic agents has been explored in recent years, including memantine, riluzole, N-acetylcysteine, topiramate, and lamotrigine, which target different aspects of glutamatergic transmission [17]. Among glutamatergic agents, ketamine and esketamine have attracted particular interest due to their uniquely rapid onset of action [18]. Both ketamine and esketamine exert their primary effects through non-competitive antagonism of the NMDA receptor. They trigger a cascade of neurobiological processes that extends beyond acute neurotransmitter modulation. NMDA receptor antagonism, particularly on inhibitory GABAergic interneurons, leads to disinhibition of pyramidal neurons and a transient surge in extracellular glutamate. This favors AMPA receptor activation and increases the AMPA/NMDA ratio. The resulting AMPA activation drives downstream signaling pathways, including BDNF release and mTOR activation [19]. These effects are associated with rapid synaptic plasticity, including increased dendritic spine formation and enhanced synaptic connectivity. In OCD, dysfunction of the CSTC circuit is a key feature, partly driven by NMDA receptor-mediated glutamatergic dysregulation. Enhancing synaptic plasticity within this circuit may help normalize network activity and glutamatergic transmission and reduce obsessive–compulsive symptoms [17]. Inhibition of AMPA receptors has been shown to prevent these effects, which highlights their necessity for ketamine and esketamine’s therapeutic actions [20].

At the molecular level, ketamine rapidly increases BDNF translation and synaptic protein expression through mTOR-dependent mechanisms. This contributes to enhanced synaptogenesis in the prefrontal cortex. This process in mediated by the inhibition of the eukaryotic elongation factor 2 (eEF2) kinase, which leads to rapid desuppression of BDNF synthesis. BDNF signaling appears to be necessary for the downstream synaptic and behavioral effects of ketamine [20,21]. Human studies suggest parallel peripheral changes in BDNF levels after ketamine administration. The clinical response was positively correlated with post-treatment levels of BDNF [22,23].

Connectivity during and after ketamine infusion has been widely studied using neuroimaging techniques. Although ketamine has been demonstrated to increase prefrontal connectivity, these changes have not been consistently associated with clinical response. Both increases and decreases in connectivity across different regions and circuits have been reported. This suggests a network-level reorganization rather than a uniform global increase in connectivity [24,25].

Encouraged by their established benefits for depression, where glutamatergic dysregulation and impaired synaptic plasticity contribute to altered network connectivity, several studies have investigated their potential efficacy in the treatment of refractory OCD. Similarly, OCD is characterized by glutamatergic abnormalities and CSTC circuit dysfunction, which are implicated in the persistence of intrusive thoughts and compulsive behaviors. The engagement of glutamatergic pathways in OCD provides a clear neurobiological rationale for ketamine and esketamine as promising bridging treatments in acute clinical states.

The aim of this scoping review is to map out and synthesize the existing evidence regarding the potential therapeutic role of ketamine or esketamine in the treatment of OCD. The particular focus of this review is to integrate preclinical and clinical findings, comparing routes of administration and addressing translational relevance. To our knowledge, these aspects have not been comprehensively synthesized within a single framework. By doing so, this review aims to identify existing gaps in the literature and provide directions for future research.

## 2. Results

**Figure 1:**
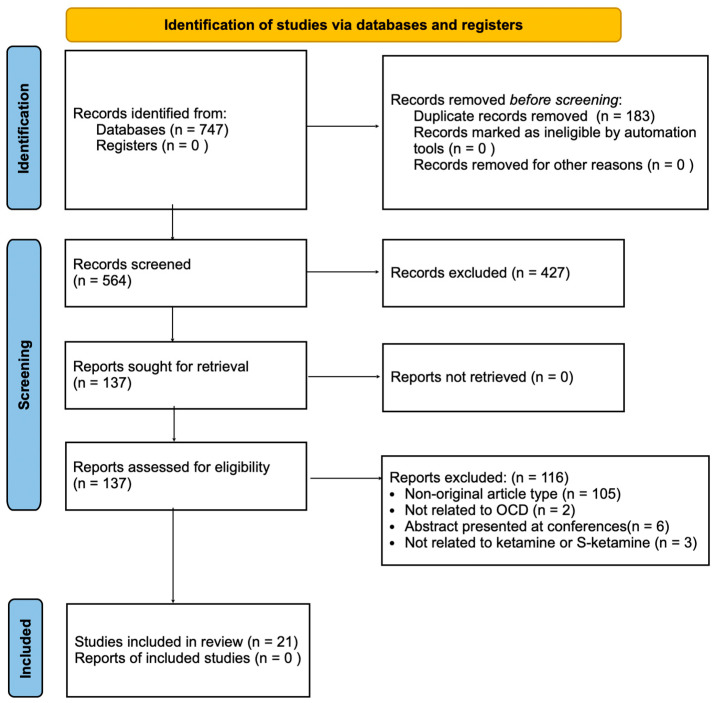
PRISMA flow diagram of study selection.

A total of 747 records were identified through database searches. After automatic duplicate removal, 564 records were screened by title and abstract, of which 137 articles underwent full-text assessments. Ultimately, 21 studies met the eligibility criteria and were included in the final analysis (see Figure 1).

### 2.1. Preclinical Evidence

Among the 21 articles included in this review, five were preclinical experimental investigations conducted on rodent models. All studies employed control between-group designs and typically used intraperitoneal saline infusions as placebo.

Across these studies, distinct OCD animal models were employed. Thompson et al. (2020) used RU24969 (a serotonergic agonist with predominant 5-HT_1_B activity that produces perseverative hyperlocomotion) [26] while Gattuso et al. (2023) relied on a genetic OCD model—Sapap3 knockout mouse, characterized by compulsive grooming and anxiety-like behaviors [27]. Other researchers used the MBT as a behavioral proxy for compulsivity [28]. Davis et al. (2021) employed a compulsivity-relevant rodent model to examine the ketamine-induced modulation of frontostriatal circuitry and repetitive behaviors [29]. Ayub et al. (2022) investigated the anti-compulsive effect of S-ketamine, both as monotherapy and as an adjuvant to fluoxetine, in Swiss mice with assessment through the MBT, OFT, and NBT [30]. Most studies primarily investigated ketamine [26,27,29], while only one study focused exclusively on S-ketamine [28]. Similarly, Ayub et al. (2022) centered their investigation on S-ketamine, administered acutely or chronically across a range of doses, alone or in combination with fluoxetine [30].

Low-dose ketamine mitigated RU24969-induced hyperlocomotion, although higher doses determined mixed outcomes over time [26]. In the Sapap3 knockout model, ketamine failed to attenuate compulsive grooming or anxiety, whereas MK-801 (a non-competitive NMDA receptor antagonist) suggested genotype-dependent NMDA receptor alterations [27]. Moreover, S-ketamine reduced marble burying behavior, and this effect was proven to be abolished by AMPA receptor blockade (through several pretreatments).

This result emphasizes the importance of AMPA-mediated plasticity rather than NMDA inhibition per se [28]. In line with these findings, Ayub et al. (2022) reported an anti-compulsive effect of S-ketamine, with notable sex differences, as higher doses were required in female mice compared with males [30]. There are some key differences between genetic and pharmacological models of OCD that should be noted. Sapap3 knockout models show high construct validity and reproduce key features of corticostriatal circuit dysfunction. Pharmacological models are more variable and may better capture acute symptom modulation rather than the underlying pathology. Detailed comparative information across studies is presented in Table 1.

Overall, the preclinical findings suggest that ketamine-related effects on compulsive-like behaviors are highly model-dependent, with more consistent responses observed in pharmacological paradigms than in genetic models. Treatment effects are influenced by dosing regimen, duration of administration, and biological variables such as sex. At a mechanistic level, the results converge on the role of downstream glutamatergic signaling, particularly AMPA receptor-mediated plasticity, rather than NMDA receptor blockade alone. These discrepancies across models underscore important translational limitations, as acute behavioral changes observed in animal paradigms may not fully capture the complexity of OCD pathophysiology in humans.

The risk of bias assessment using the SYRCLE tool showed low methodological reporting quality across preclinical studies, with insufficient reporting of selection, performance, and detection biases, particularly regarding randomization and blinding. Attrition bias was better addressed, while reporting bias remained unclear due to limited transparency [31]. A detailed critical appraisal is provided in the Supplementary Materials.

### 2.2. Clinical Evidence

#### 2.2.1. Randomized Clinical Trials

- Active-controlled/psychoactive controlled

The two active-controlled RCTs identified began with a similar study design. However, they differed significantly in administration route and active control choice. Beaglehole et al. (2024) [32] investigated IM ketamine injections controlled with IM fentanyl and managed to enroll and maintain the engagement of most of its participants (10 out of 12 patients completed the entire protocol). Both doses outperformed fentanyl in efficacy. Sixty percent of patients met treatment response after 0.5 mg/kg ketamine injections, but higher concentrations did not show any additional benefit [32]. By contrast, the study conducted by Rodriguez et al. (2017) [33], which used intranasal midazolam as a control, was discontinued prematurely due to poor participant recruitment (only two patients recruited out of the 20 enrolled) and suboptimal tolerability. Neither participant met the response criteria [33]. Further comparative details are provided in Table 2. These findings suggest that while ketamine may demonstrate clinically meaningful effects in controlled settings, the evidence remains limited and highly sensitive to study design factors such as administration route, comparator choice, and feasibility of recruitment. Notably, the very low retention and the premature discontinuation of one trial substantially limit its statistical power. This outcome raises concerns regarding the robustness of its protocol, including eligibility criteria and overall study feasibility.

- Placebo-controlled

Both placebo-controlled studies used vehicle controls (saline IV infusions or nasal spray) and involved participants that received both ketamine (0.5 mg/kg IV over 40 min) and saline, separated by at least one week. The population samples were highly homogenous: unmedicated adults with moderate or severe OCD, mean Y-BOCS scores of 26–28, a long course of illness (17–18 years), minimal comorbid depression (HDRS < 25), and prior failure or refusal of SSRIs and/or CBT-ERP [34,35].

Rodriguez et al. (2013) [35] evaluated clinical, acute and short-term outcomes in obsessive symptoms severity following ketamine infusions compared to placebo. The researchers used the OCD-VAS and the Y-BOCS to assess outcomes. Significant carryover effects imposed the restriction of analysis to first-phase data only, which reduced the effective sample size [35] (see Table 3).

Rodriguez et al. (2015) [34] conducted a neuroimaging study and used a largely overlapping sample and focused on prefrontal neurochemical outcomes. Both infusion periods were analyzed, as no evidence of neurochemical carryover effects was observed. Neurochemical changes were assessed using proton magnetic resonance spectroscopy of the MPFC and measured GABA/W and Glx/W concentrations [34]. These metabolite levels were subsequently correlated with changes in clinical symptoms (see Table 3).

Overall, in these two studies, ketamine was associated with rapid anti-obsessional effects and with transient GABA increases in the MPFC, respectively. Both studies provide preliminary evidence for clinical and neurobiological effects of ketamine in OCD.

These studies suggest that ketamine produces rapid anti-obsessional effects alongside measurable neurochemical changes in prefrontal regions. However, the strength of this evidence is constrained by small sample sizes, overlapping cohorts and methodological limitations such as carryover effects that reduce statistical robustness.

The critical appraisal using the JBI-appropriate tool indicated moderate methodological quality. Randomization was adequate, but allocation concealment and baseline comparability were often unclear. Blinding was inconsistently reported. Outcome assessment was generally robust and statistical analyses were appropriate. However, reporting of participant handling and study procedures was sometimes insufficient [36]. A detailed appraisal is provided in the Supplementary Materials.

#### 2.2.2. Open-Label Studies

Both single-infusion and multiple-infusion ketamine protocols have been investigated [37,38]. After the single infusion strategy, symptoms statistically improved within 3 days post-infusion but none of the patients met the criteria for treatment response [38]. Multiple infusions managed to achieve response in one patient and partial response in two others [37]. In a recent study, Beaglehole et al. (2025) [39] used oral ketamine as maintenance treatment after treatment response was achieved with IM ketamine injections in a previous phase. The reduction from the prior phase was sustained over 6 weeks of oral ketamine maintenance, which was considered as the treatment response [39]. Detailed information regarding the study protocols, sample characteristics, and outcomes can be found in Table 4.

These findings suggest that while single infusions may produce rapid symptom improvements, repeated or maintenance strategies may be necessary to achieve and sustain clinically meaningful responses. The limited number of responders and small sample sizes across studies restrict the strength of these conclusions and highlight the need for more robust evidence.

The methodological quality assessment indicated moderate study rigor. All studies supported a clear cause–effect relationship, but the absence of control groups increases the risk of selection bias. Outcome assessment and statistical analyses were appropriate, while follow-up reporting was often insufficient [40]. A detailed appraisal is provided in the Supplementary Materials.

#### 2.2.3. Case Series

Case series reveal inconsistent response rates. While some patients exhibited sustained symptom relief following repeated ketamine infusions, others showed minimal benefit or experienced significant adverse effects.

Two studies investigated IV ketamine in adolescents with treatment-refractory OCD [41,42]. In the case series presented by Kumar et al. (2025) [41], although treatment response was not explicitly defined, the three participants achieved important reductions in CY-BOCS scores and the benefit persisted up to 2 months. However, improvement emerged only after repeated infusions, which suggests the need for repeated dosing for sustained anti-obsessional effects. Psychosis occurred in the youngest patient included in this case series, which might suggest a potential age-dependent vulnerability [41]. In contrast, Ishimuro et al. (2025) observed rapid reduction in OCD severity following ketamine infusions in adolescents, including for patients with multiple psychiatric comorbidities [42]. The key methodological differences between the two studies are drug administration frequency and follow-up duration. Kumar et al. (2025) used multiple ketamine infusions and assessed outcomes up to 2 months [41], whereas Ishimuro et al. (2025) used a single infusion protocol with a shorter follow-up period [42]. Detailed information regarding the study protocols, sample characteristics, and outcomes can be found in Table 5.

As a follow-up to the open-label trial conducted by Bloch et al. (2012) [38], Niciu et al. (2013) reported two patients with OCD who developed delayed dysphoria, worsening anxiety, and suicidal ideation after ketamine infusion, despite minimal depressive symptoms at baseline [43].

Differences in outcomes between the case series appear to be driven in part by variations in dosing strategy and follow-up duration. Repeated infusion protocols show more sustained effects, while single infusions are associated with rapid but less durable responses. It is important to note that the inclusion of adolescent populations introduces additional heterogeneity, as neurodevelopmental differences and distinct clinical characteristics may limit the direct comparability of these findings with adult OCD populations.

The JBI methodological quality appraisal indicated moderate methodological quality. Eligibility criteria and outcome measures were generally well-defined. Recruitment processes were often unclear, suggesting potential selection bias. Demographic data, outcomes, and follow-up were generally well reported [44]. Further details are available in the Supplementary Materials.

#### 2.2.4. Case Reports

Kaltenboeck et al. (2023) [45] reported a 28-year-old man with severe, TR-OCD (Y-BOCS > 35, CGI-OCD 7) and severe comorbid depression who failed to respond to multiple prior therapeutic strategies. He received 10 IV esketamine infusions (0.35 mg/kg) with concurrent psychotherapy over 2 months, followed by intranasal esketamine maintenance (two weekly sessions with 0.8–1.2 mg/kg esketamine) and outpatient intranasal esketamine every 4–6 weeks alongside CBT and pharmacotherapy (sertraline, pregabalin, sodium valproate). After 2 months, OCD and depression symptoms consistently improved. At 10-week follow-up, depression remitted and OCD further improved, which indicates sustained clinical benefit [45].

Rodriguez et al. (2011) [46] presented the case of a 24-year-old woman with severe, TR-OCD (Y-BOCS 30), minimal depression and no other psychiatric comorbidities, who was non-responsive to three prior SSRI trials and augmentation strategies. She received two ketamine IV infusions of 0.5 mg/kg over 40 min (with saline as control). During the second ketamine infusion, her obsessions ceased completely, partially returned 40–230 min after the infusion, but did not fully return until day 7 post-infusion. Vital signs remained stable, and dissociative symptoms were mild and transient [46].

Veraart et al. (2021) [47] reported a 55-year-old woman with severe, TRD comorbid with OCD and psychotic symptoms. She received oral esketamine (0.5 mg/kg twice weekly, titrated up to 2.0 mg/kg) alongside DBS and conventional treatment (venlafaxine, clozapine, glycopyrronium, movicolon, nitrazepam). At the 6-week follow-up, depression improved significantly, with functional gains and reduction in both auditory hallucinations and OCD symptoms [47].

Matteo et al. (2021) [48] reported a 39-year-old male with TRD and comorbid OCD, treated with venlafaxine, bupropion, lamotrigine and olanzapine, who received intranasal esketamine following a standard induction and maintenance scheme. Esketamine led to rapid improvement of depressive symptoms during induction and significant reduction in OCD symptoms during maintenance, with marked clinical improvement and functional recovery. By the ninth administration, olanzapine and lamotrigine were tapered off. Residual anxiety was managed with pregabalin 225 mg/day [48].

A more recent case report by Algin et al. (2024) [49] described a 24-year-old man with TR-OCD and severe suicidal ideation. He received IV ketamine (0.5 mg/kg over 60 min) once a week for 3 weeks. After the first infusion, his OCD symptoms and suicidal ideation showed rapid but transient improvement. Suicidal ideation returned temporarily due to a stressful life event. Following the second infusion, his symptoms improved substantially. After the third infusion, OCD, depressive, and suicidal symptoms remained at subclinical levels, and these improvements were sustained at 6- and 12-week follow-ups. Treatment was well tolerated, with only transient drowsiness observed [49].

Adams et al. (2017) [50] reported the case of a male patient in his late 20s with severe, TR-OCD and comorbid depression with chronic suicidal ideation. He received intensive inpatient CBT for 8 weeks and outpatient CBT for 8 additional weeks, with twice-weekly intranasal ketamine (50 mg per session, 5 × 10 mg doses over 20 min) administered during weeks 3–6 of inpatient treatment. Ketamine was well tolerated, producing mild, transient dissociative effects, and vital signs remained stable. OCD symptoms improved after the first week, with improvements maintained through inpatient treatment. Post-discharge, symptoms initially worsened but gradually improved with continued outpatient CBT. Ketamine appeared to accelerate OCD symptom reduction, enhance CBT-ERP engagement, and rapidly reduce suicidality. The independent effect of CBT-ERP could not be isolated [50].

Compared to controlled studies’ patients, case reports are characterized by greater clinical complexity, with frequent use of concurrent interventions such as CBT-ERP, DBS, and multi-drug regimens. This makes treatment effects less attributable to ketamine alone but also suggests a potential role of ketamine or esketamine as adjunctive strategies in highly refractory cases. These agents may be particularly relevant for enhancing psychotherapy engagement and targeting comorbid symptoms such as depression and suicidality.

The JBI critical appraisal of the included case reports indicates an overall high reporting quality, with strong clinical detail and only minor limitations in reporting diagnostics, adverse events, or demographics [51]. Additional details of the critical methodological appraisal are provided in the Supplementary Materials.

## 3. Discussion

The current body of literature on ketamine and esketamine in OCD is marked by considerable clinical and methodological heterogeneity.

Although the preclinical studies differ considerably in their experimental design, they collectively contribute valuable insights into the neurobiological dimensions of OCD and the potential role of ketamine and esketamine in its treatment. Despite these differences, all studies explored pathways converging on the glutamatergic signaling (whether through NMDA receptor antagonism, AMPA receptor modulation or mGluR5 related alterations) with effects on frontostriatal compulsivity. Each study provides complementary perspectives on the interplay between NMDA, AMPA, and mGluR5 receptor systems, the key components of the glutamate network that is implicated in the OCD pathophysiology. Ketamine and esketamine appear to act primarily through modulation of the excitatory transmission while dose, timing, and receptor context shape the outcomes. These effects are also reflected in downstream pathways involving BDNF and mTOR, which contribute to synaptic plasticity and circuit-level changes. In preclinical models, modulation of these pathways is associated with the normalization of frontostriatal activity and reduction of compulsive-like behaviors which link the molecular effects to observed behavioral outcomes.

While preclinical models provide essential insights into the neurobiology of OCD, their translational validity remains limited. Most paradigms focus on repetitive or compulsive-like behaviors, which only partially reflect the clinical heterogeneity and cognitive complexity of the disorder. Core features such as obsessions, intrusive thoughts, and subjective distress are generally overlooked. Interspecies differences in neurocircuitry and the controlled nature of laboratory environments further constrain direct extrapolation. These limitations underscore the need for integrative approaches combining preclinical data with clinical and neuroimaging studies to improve translational validity.

The clinical studies differ substantially regarding patient characteristics, definition of treatment resistance, intervention protocol, and outcome assessment, which limit direct comparisons and make quantitative analysis impossible. Key differences and salient findings have been systematically identified and discussed across studies, in line with the exploratory and mapping objectives inherent to a scoping review.

### 3.1. Treatment Resistance

While most studies focused on TR-OCD, the criteria used to define treatment resistance varied considerably. Some applied a more stringent definition and required failure of at least two pharmacological treatments in combination with at least one course of CBT [32,38,39,41,42], while others defined TR-OCD as failure to respond to at least one adequate SSRI trial or CBT intervention [34]. In some cases, the sample was heterogeneous and most participants had failed two medications. However, no stringent inclusion criteria were applied, and treatment resistance was loosely defined [37]. As a result, studies that focused on treatment-resistant patients likely differed substantially with regard to sample characteristics.

### 3.2. OCD Severity

Baseline OCD severity also varied across studies. About half of them required severe OCD, as determined by Y-BOCS scores [32,38,41,45,46,49,50], whereas the others also allowed patients with moderate symptom severity to be included [33,34,35]. Importantly, several studies that included moderately ill participants did not require treatment resistance as an inclusion criterion, which further contributes to sample variability. Some studies did not specify a criterion for OCD severity, but participants were generally severely affected based on the mean Y-BOCS scores reported [37,39,42]. However, participants were selected by the criterion of near-constant obsessions, which enhances the sensitivity to immediate symptom change but limits generalizability to the broader OCD population.

### 3.3. Comorbidity

Handling of psychiatric comorbidities, particularly depression, differed substantially between studies. Some studies explicitly excluded patients with moderate or severe comorbid depression [32], whereas others either only excluded patients with severe depression [33,34,35,39] or accepted comorbid depression [37,38,41,42]. Given ketamine’s established antidepressant effects, this difference may have influenced observed outcomes.

### 3.4. Medication Status

Medication status at baseline further distinguished study populations. Some study protocols allowed participants to continue their existing oral psychotropic medications during ketamine administration [32,33,37,38,39,41,42], whereas others required participants to be withdrawn from all psychiatric oral medications prior to intervention [34,35]. As a result, ketamine was administered as augmentation in some studies and as monotherapy in others, making direct comparison of outcomes impossible.

### 3.5. Intervention Protocol

Differences in routes of administration may reflect distinct pharmacokinetic profiles. IV administration provides rapid peak plasma levels, ensures rapid onset and precise dosing. Intranasal and oral routes are associated with slower absorption and greater interindividual variability [52]. These differences may influence onset, efficacy, and tolerability, and should be considered when comparing outcomes across studies.

Most trials administered IV ketamine (0.5 mg/kg) [34,35,37,38,41,42] with alternative routes including IM [32], intranasal [33], and oral [39]. Protocols varied between multiple administrations at different frequencies [32,37,39,41] and single time administrations [33,34,35,38,42]. Beyond the route of administration, these distinct therapeutic strategies make any generalization inadequate.

Intravenous administration appears to be associated with more rapid and consistent clinical effects, likely due to its predictable pharmacokinetic profile, whereas intranasal and oral routes may result in greater variability in response. Repeated dosing may be associated with more sustained symptom improvement compared to single-dose interventions, although evidence remains limited and heterogeneous.

### 3.6. Response Definition

Most studies defined clinical response as a reduction of at least 35% in Y-BOCS scores [33,34,35,37,38,39]. Some investigators applied a more stringent threshold requiring a 50% reduction in OCD scores [32] while others did not clearly specify a treatment response threshold [41,42].

### 3.7. Study Design and Control Conditions

Regarding the actively controlled trials, the crossover design used by Beaglehole et al. (2024) [32] was found to be unsuitable due to the significant persistence of ketamine’s effects beyond the wash-out period. This factor likely influenced the results obtained during fentanyl administration in the group initially assigned to ketamine. Moreover, fentanyl functioned poorly as an active control, since participants received both interventions. This aspect allowed acute psychoactive differences to be easily recognized thus blinding may have been potentially compromised. The vehicle-controlled studies by Rodriguez et al. (2015) and Rodriguez et al. (2013) were unable to maintain effective blinding due to ketamine’s distinctive psychoactive effects, which inherently compromises the validity of the control condition [34,35].

A notable strength of the study conducted by Beaglehole et al. (2024) was the premedication with ondansetron and the dose adjustment after poor tolerability at higher ketamine doses, which probably helped minimize dropouts and improve participant comfort [32].

A notably high dropout rate was observed in the Rodriguez et al. (2017) [33] trial, primarily driven by refusal of intranasal administration. This contrasts with depression trials, where intranasal administration is generally better accepted. This finding may suggest that intranasal delivery is less feasible in OCD populations, potentially related to contamination concerns or sensory sensitivities commonly observed in this disorder [33].

### 3.8. Population Characteristics

Inclusion criteria varied considerably regarding age, number of prior treatments, comorbidities, and baseline OCD severity. Therefore, comparisons between studies should be interpreted with caution, as outcomes are likely influenced by these differences in patient populations.

### 3.9. Outcome Assessment and Follow-Up

Most trials focused primarily on obsessive symptoms, particularly those that used single-dose administrations and short-term follow-ups. This approach limits evaluation of the compulsive dimension of OCD. Studies with longer follow-up periods and multiple administrations over time are more likely to achieve an accurate representation of potential improvements in overall OCD symptomatology. One trial adapted the Y-BOCS for short-term scaling to capture symptom changes within the first week post-administration [32]. Compulsions are difficult to assess during and immediately after intervention, as hospital settings often lack relevant triggers, and behaviors may be suppressed by ketamine’s acute psychoactive effects.

### 3.10. Treatment Response

Meaningful symptom improvement was observed in several investigations, with response rates of 50–63% [32,35]. In contrast, in the other studies, no participants met the criteria for response, and only minimal or no clinical improvement was observed [33,38]. Limited efficacy was also reported in some samples, as most participants failed to respond and only isolated cases demonstrated partial or full response [37].

Across individual case reports, ketamine or esketamine administration is consistently associated with fast onset and clinically significant reduction in obsessive–compulsive symptoms, particularly in patients with treatment-resistant OCD and comorbid depression or suicidality. In these cases, reduction in Y-BOCS scores and suicidal ideation were observed shortly after infusion, with effects that lasted from days to weeks [29,45,46,49].

While preclinical studies consistently demonstrated anti-compulsive effects, clinical findings are more variable, which highlights a translational gap between experimental models and human OCD. Across clinical studies, a consistent pattern emerges of rapid symptom reduction following ketamine administration, with effects typically observed within hours to days, but is often transient in duration. Higher doses and intravenous delivery were generally associated with more robust but short-lived effects. The most common approach was a single ketamine administration. However, the few studies that used repeated administrations appeared to achieve better response rates.

Taken together, these findings suggest that the variability in reported outcomes is more likely attributable to differences in patient selection and characteristics, intervention type and protocol, methodological rigor and study designs, and should not be considered fundamentally contradictory evidence regarding ketamine’s therapeutic potential in OCD. Potential clinical benefits were most consistently observed in studies that used intravenous ketamine in patients with moderate symptom severity and less restrictive criteria for treatment resistance [32,35]. Studies that focused on more severely symptomatic patients and defined treatment resistance more strictly generally demonstrated limited or no clinical improvement [37,38]. It is important to note that adjunctive therapeutic strategies, such as exposure-based CBT or ketamine use as a maintenance strategy, also achieved a better clinical response. This may suggest that ketamine may act as a facilitator of other therapeutic interventions rather than as a monotherapy option [37,39]. Current literature provides preliminary signals rather than definitive evidence and underscores the need for larger, standardized, and adequately controlled trials.

### 3.11. Limitations

Interpretation of the current evidence is constrained by several important limitations. The clinical studies included in this review demonstrate significant heterogeneity in inclusion criteria, particularly regarding age, treatment history, psychiatric comorbidities, and baseline OCD severity. Intervention protocols were also highly inconsistent, with variations in dosing, frequency of administration, and route of delivery. Differences in how treatment resistance was conceptualized further complicate comparisons across samples. Blinding was often compromised by ketamine’s recognizable psychoactive effects, and follow-up periods were frequently short. The broad inclusion criteria of this review, encompassing both preclinical and clinical studies as well as diverse study designs, may have further increased heterogeneity and limited direct comparability between findings.

## 4. Materials and Methods

The methodological approach was guided by the framework proposed by Arksey and O’Malley (2005) [53] and further refined by the JBI guidelines [54]. The review process was reported following the PRISMA-ScR checklist to ensure transparency and reproducibility [55] (see Supplementary Materials).

The search was conducted across three major electronic databases: PubMed, Scopus, and Web of Science, covering all available literature regarding the use of ketamine or esketamine in OCD patients or relevant preclinical models for OCD. Both preclinical and clinical studies were included to support a translational approach. This approach allowed the integration of mechanistic insights from experimental models with clinical data on efficacy and safety. This enabled a more complete understanding of the link between OCD pathophysiology and therapeutic clinical potential. The search was conducted on the 20th of October 2025 without any restrictions on language, publication date, or study design to ensure comprehensive coverage of the evidence base. We used the following keywords, along with Boolean operators AND/OR: (Obsessive–Compulsive Disorder, OR OCD OR obsession OR compulsion) AND (Esketamine OR Ketamine). A complete, reproducible search strategy for each database, including full search strings, is provided in the Supplementary Materials. All retrieved records were imported into Rayyan software (www.rayyan.ai, first accessed on 20 October 2025) and duplicates were removed prior to screening [56]. The selection process based on the title and abstract was carried out independently by two reviewers (M.M. (Maria Marmureanu) and S.I.V.), followed by full-text assessments for potentially eligible studies. If consensus was not reached during title and abstract screening or full-text review stages, a third reviewer was consulted (V.D.). Eligibility was determined based on predefined inclusion and exclusion criteria, applied consistently by both reviewers. The eligibility criteria were structured according to the Population–Concept–Context framework (see Table 6). Methodological quality and risk of bias were assessed using the JBI critical appraisal checklists for clinical studies (including RCTs, quasi-experimental studies, case series and case reports) and the SYRCLE risk-of-bias tool for preclinical studies [31,36,40,44,51]. Detailed results of the critical appraisal are provided in the Supplementary Materials.

The data from the included studies were systematically charted using a structured template developed to ensure consistent documentation of study design, patient and model characteristics, intervention details, outcome measures, and key findings. Extracted data were cross-checked by both reviewers to ensure accuracy and consistency.

## 5. Conclusions

Taken collectively, the reviewed studies suggest that modulation of the glutamatergic system may represent a promising therapeutic target in OCD. Ketamine and esketamine appear to produce rapid symptom improvement in some patients, particularly those with TR-OCD, although these effects are often transient and not consistently observed across studies. Preclinical findings further support the role of NMDA, AMPA, and mGluR5 signaling in the neurobiology of obsessive–compulsive symptomatology, but their translation into sustained clinical benefit remains uncertain. Considerable heterogeneity across studies in patient selection, intervention protocols, and methodological design limits the strength of current conclusions, particularly regarding optimal dosing strategies, routes of administration, and patient subgroups most likely to benefit. Future research should prioritize larger randomized trials, direct comparisons of administration routes and dosing regimens, standardized definitions of treatment resistance, and longer follow-up periods to determine the durability and clinical relevance of these effects. This review highlights the need for more standardized and translationally aligned research approaches.

## Figures and Tables

**Table 1:** Preclinical studies: comparative analysis of ketamine and esketamine effects.

Article	Ayub et al. (2022) [30]	Thompson et al. (2020) [26]	Gattuso et al. (2023) [27]	Tosta et al. (2019) [28]	Davis et al. (2021) [29]
Tested agent	Esketamine ± fluoxetine	Ketamine	Ketamine MK-801	Esketamine	Ketamine
Animal model	Swiss mice	RU24969 challenge	Sapap3 knockout (KO)	Naive Swiss mice	SAPAP3 knockout mouse
Intervention protocol	Esketamine (IP, 3–30 mg/kg; acute/chronic) ± fluoxetine (2.5–10 mg/kg)	Ketamine (IP, 0–30 mg/kg; single, pre-RU24969)	Ketamine (IP, 30 mg/kg)/MK-801 (IP, 0.25 mg/kg; single)	Esketamine (IP, 3–30 mg/kg)/vmOFC (0.3–10 nmol)	Ketamine (IP, 30 mg/kg)/saline (IP; single)
Comparator	Fluoxetine (10 mg/kg)	Saline (pre-/post-treatment)	Saline; WT vs. Sapap3 KO	Saline; fluoxetine (10 mg/kg, positive control)	WT mice
Assessment	MBT OFT NBT	Hyperlocomotion, PPI (post-RU24969)	Light-dark box, grooming, locomotion, PPI.	MBT OFT	Grooming (bouts, time, frequency), locomotion
Results	Transient, dose- and sex-dependent anti-compulsive effects; synergy with fluoxetine; no influence of sex hormones.	Low dose: delayed benefit; high dose: transient worsening.	Sapap3 KO mice: increased anxiety and grooming, no ketamine response, altered MK-801 effects.	Dose-dependent effects, higher doses effective; only intra-vmOFC S-ketamine was effective.	Reduced grooming frequency and total grooming time in Sapap3 KO mice.

Abbreviations: MK-801, dizocilpine maleate, a non-competitive NMDA receptor antagonist; RU24969, serotonin receptor agonist with predominant 5-HT_1_B activity; SAPAP3, synapse-associated protein 90/postsynaptic density-95-associated protein 3; IP, intraperitoneally; WT, wild type; KO, knockout; MBT, marble burying test; OFT, open field test; NBT, nest building test; PPI, prepulse inhibition; vmOFC, ventromedial orbitofrontal cortex.

**Table 2:** Clinical evidence: randomized, actively controlled trials.

Article	Beaglehole et al. (2024) [32]	Rodriguez et al. (2017) [33]
Tested agent	Ketamine	Ketamine
Sample	12 participants randomized; 10 completed all arms (7 female, 3 male)	2 participants completed the protocol before discontinuation (out of 20 eligible).
OCD severity	Severe YBOCS > 26	At least moderate YBOCS > 16
Comorbidity	Patients with moderate–severe comorbid depression were excluded (MADRS > 20)	Patients with severe comorbid depression were excluded (MADRS > 25)
Treatment resistance	TR (failure of ≥2 pharmacological and ≥1 psychotherapeutic interventions)	Not specified
Medication status	Participants treated with psychotropic medication	Participants treated with psychotropic medication
Intervention protocol	IM racemic ketamine 0.5–1.0 mg/kg, three sessions spaced ≥1 week apart + 4 mg oral ondansetron 1 h prior to dosing	Intranasal ketamine, single 50 mg administration
Comparator	Fentanyl 50 μg	Intranasal midazolam 4 mg
Response definition	>50% score reduction at 24 h compared to baseline	≥35% Y-BOCS reduction at 1 week.
Outcome measures	Y-BOCS at baseline, 1 h, 2 h, 24 h, 168 h; CADSS; BPIC-SS, blood pressure, adverse events	Y-BOCS; HDRS-17; adverse effects/tolerability during and after administration
Main results	OCD symptoms improved with both doses versus no change with fentanyl; 0.5 mg/kg most effective (60% responders); 1 mg/kg caused more dissociation and dropouts	No participants met response criteria; minimal Y-BOCS/HDRS changes; intranasal route poorly tolerated (burning, stinging, bitter taste)

Abbreviations: Y-BOCS, Yale–Brown Obsessive–Compulsive Scale; MADRS, Montgomery–Åsberg Depression Rating Scale; TR, treatment-resistant; IM, intramuscular; CADSS, Clinician-Administered Dissociative States Scale; BPIC-SS, Brief Psychiatric Rating Scale–Suicidality Subscale; HDRS-17, 17-Item Hamilton Depression Rating Scale.

**Table 3:** Clinical evidence: randomized, placebo/vehicle-controlled trials.

Article	Rodriguez et al. (2015) [34]	Rodriguez et al. (2013) [35]
Tested agent	Ketamine	Ketamine
Sample	17 enrolled, 16 analyzed (1 excluded for outlier MRS data)	15 randomized (8 ketamine first, 7 saline first); all completed both infusions
OCD severity	At least moderate YBOCS > 16	At least moderate YBOCS > 16
Comorbidity	Patients with severe comorbid depression were excluded (HDRS > 25); some psychiatric comorbidities were allowed	Patients with severe comorbid depression were excluded (HDRS > 25)
Treatment resistance	Treatment-resistant (failed ≥1 SSRI and/or CBT-ERP, or refused treatments)	Treatment-resistant (failed ≥1 SSRI and/or CBT-ERP, or refused these treatments)
Medication status	Medication-free at baseline	Medication-free at baseline
Intervention protocol	IV ketamine, single subanesthetic 0.5 mg/kg infusion over 40 min (during MRI)	IV ketamine, single subanesthetic 0.5 mg/kg infusion over 40 min
Comparator	Placebo control (saline infusion)	Placebo control (saline infusion)
Response definition	Not specified	≥35% Y-BOCS reduction
Outcome measures	OCD-VAS, Y-BOCS, HDRS-17; MRI + ^1^H-MRS (MPFC: GABA/W, Glx/W); correlations (OCD-VAS, dissociative/psychotic scales)	OCD-VAS; Y-BOCS; HDRS-17; CADSS
Main results	Ketamine transiently increases MPFC GABA (not glutamate); GABA rise correlates with symptom improvement and suggests GABAergic involvement in anti-OCD effect	Rapid, significant obsessions reduction during infusion, lasting up to 1 week; 50% met ≥35% Y-BOCS reduction vs. 0% placebo; HDRS-17 unchanged

Abbreviations: Y-BOCS, Yale–Brown Obsessive–Compulsive Scale; HDRS, Hamilton Depression Rating Scale; CBT-ERP, cognitive-behavioral therapy with exposure and response prevention; MRI, magnetic resonance imaging; IV, intravenous; OCD, Obsessive-Compulsive Disorder; OCD-VAS, Obsessive–Compulsive Disorder Visual Analog Scale; H-MRS, proton magnetic resonance spectroscopy; MPFC, medial prefrontal cortex; GABA/W, gamma-aminobutyric acid normalized to water; Glx/W, glutamate + glutamine normalized to water; GABA, gamma-aminobutyric acid; CADSS, Clinician-Administered Dissociative States Scale; SSRI, selective serotonin reuptake inhibitor.

**Table 4:** Clinical evidence: open-label studies.

Article	Bloch et al. (2012) [38]	Beaglehole et al. (2025) [39]	Sharma et al. (2020) [37]
Tested agent	Ketamine	Ketamine	Ketamine
Sample	10 enrolled; 100% completed	8 enrolled; 5 completed (6 weeks)	14 participants
OCD severity	Severe YBOCS > 24	Severe (mean YBOCS 30.4)	Severe (mean YBOCS 31)
Comorbidity	Comorbid depression allowed	Severe depression excluded (HDRS > 25)	Comorbid depression allowed
Treatment resistance	TR (≥2 pharmacological treatment ± CBT)	TR (≥2 pharmacological treatment + ≥1 psychotherapy)	TR (≥2 trials; majority CBT non-responders)
Medication status	Mixed (3 unmedicated; SSRIs ± antipsychotics)	On psychotropic medication	On psychotropic medication
Intervention protocol	IV ketamine (0.5 mg/kg, 40 min; single)	Oral ketamine (1–2 mg/kg, 1–3×/week, 6 weeks)	IV ketamine (0.5 mg/kg, 2–3×/week; 2–10 infusions)
Response definition	≥35% Y-BOCS reduction (1–3 days after infusion)	Maintenance of prior response	Response ≥ 35% Y-BOCS reduction; Partial response 25–35% Y-BOCS reduction
Outcome measures	Y-BOCS; HDRS-17; CGI; CADSS	HADS-A/HADS-D, IES-R, Y-BOCS; CADSS; BPIC-SS; blood pressure	Y-BOCS; HAM-D
Main results	No response; ~12% improvement; adverse effects (dysphoria, anxiety, suicidal ideation)	~65% Y-BOCS reduction sustained (6 weeks)	1 response, 2 partial; no single-dose effect; 2 cases of depersonalization and dyspnea

Abbreviations: Y-BOCS, Yale–Brown Obsessive–Compulsive Scale; HDRS-17, 17-Item Hamilton Depression Rating Scale; TR, treatment-resistant; CBT, cognitive-behavioral therapy; SSRI, selective serotonin reuptake inhibitor; IV, intravenous; OCD, obsessive–compulsive disorder; CGI, Clinical Global Impression; CADSS, Clinician-Administered Dissociative States Scale; HADS-A, Hospital Anxiety and Depression Scale—Anxiety Subscale; HADS-D, Hospital Anxiety and Depression Scale—Depression Subscale; IES-R, Impact of Event Scale—Revised; BPIC-SS, Brief Psychiatric Rating Scale—Suicidality Subscale; HAM-D, Hamilton Depression Rating Scale.

**Table 5:** Clinical evidence: case series.

Article	Kumar et al. (2025) [41]	Ishimuro et al. (2025) [42]
Tested agent	Ketamine	Ketamine
Sample	Four adolescents (ages 13–19)	Five adolescents (Mean age 16.6 SD: 1.5)
OCD severity	Severe CY-BOCS ≥ 24	Mean baseline CY-BOCS: 29
Comorbidity	Mild depression (*n* = 2)	Anxiety (all); depression (*n* = 4); ADHD (*n* = 1)
Treatment resistance	TR (≥2 failed SSRI + CBT)	≥1 failed SSRI/clomipramine + CBT-ERP
Medication status	On psychotropic medication	Mixed (some completed washout, some on concurrent medication allowed)
Intervention protocol	IV ketamine (0.5 mg/kg, 45 min; alternate days; 5–6 infusions)	IV ketamine (0.5 mg/kg, 40 min; single)
Response definition	Not specified	Not specified
Outcome measures	CY-BOCS; CADSS.	Adverse events, vital signs, ECG, C-SSRS, CADSS, OCD-VAS, Y-BOCS, CY-BOCS, CDRS-R, CGI, client satisfaction (14 days post-infusion).
Main results	Improvement required repeated infusions; 1 case of psychosis (treatment discontinued). Responders showed ~31–49% CY-BOCS reduction, sustained at 2 months.	Rapid reduction in CY-BOCS (mean 26.2 at day 14) by 9.7% at 14 days post-infusion

Abbreviations: CY-BOCS, Children’s Yale–Brown Obsessive–Compulsive Scale; SD, standard deviation; ADHD, attention-deficit/hyperactivity disorder; SSRI, selective serotonin reuptake inhibitor; CBT, cognitive-behavioral therapy; CBT-ERP, cognitive-behavioral therapy with exposure and response prevention; IV, intravenous; CGI, Clinical Global Impression; CADSS, Clinician-Administered Dissociative States Scale; C-SSRS, Columbia–Suicide Severity Rating Scale; OCD-VAS, Obsessive–Compulsive Disorder Visual Analog Scale; Y-BOCS, Yale–Brown Obsessive–Compulsive Scale; CDRS-R, Children’s Depression Rating Scale—Revised; ECG, electrocardiogram.

**Table 6:** Inclusion and exclusion criteria for article selection according to Population–Concept–Context framework.

	Inclusion Criteria	Exclusion Criteria
Population	Studies involving patients with a formal diagnosis of OCD, regardless of age, sex or severity Preclinical investigations on animal models relevant to OCD	Studies without an OCD component
Concept	Ketamine or esketamine administered via any route (intravenous, intranasal, subcutaneous, etc.) in studies reporting original clinical or mechanistic outcomes related to OCD	Studies that evaluate other NMDA modulators or interventions Studies not reporting OCD-specific outcomes Secondary sources: review articles, meta-analyses, editorial, letters to the editor, commentaries, conference abstracts, etc.
Context	All clinical or preclinical/experimental settings, with no restrictions regarding publication year, language or country	Articles not published in peer-reviewed journals

Abbreviations: OCD, obsessive–compulsive disorder; NMDA, N-Methyl-D-Aspartate receptor.

## Data Availability

No new data were created or analyzed in this study. Data sharing is not applicable.

## Supplementary Materials

The following supporting information can be downloaded at: https://www.mdpi.com/article/10.3390/ph19040628/s1, Table S1: Preferred Reporting Items for Systematic reviews and Meta-Analyses extension for Scoping Reviews (PRISMA-ScR) Checklist; Table S2: SYRCLE Risk of Bias Assessment of Included Preclinical Studies; Table S3: JBI Critical Appraisal of Risk of Bias in Included Randomized Controlled Trials; Table S4: JBI Critical Appraisal of Risk of Bias in Included Open-Label Studies; Table S5: JBI Critical Appraisal of Risk of Bias in Included Case Series Studies; Table S6: JBI Critical Appraisal of Reporting Quality in Included Case Reports.

## Abbreviations

The following abbreviations are used in this manuscript:

ADHD	Attention-Deficit/Hyperactivity Disorder
AMPA	α-Amino-3-Hydroxy-5-Methyl-4-Isoxazolepropionic Acid Receptor
BDNF	Brain-Derived Neurotrophic Factor
BPIC-SS	Brief Psychiatric Rating Scale—Suicidality Subscale
CBT	Cognitive Behavioral Therapy
CBT-ERP	Cognitive Behavioral Therapy with Exposure and Response Prevention
CADSS	Clinician-Administered Dissociative States Scale
CDRS-R	Children’s Depression Rating Scale—Revised
CGI	Clinical Global Impression
CGI-D	Clinical Global Impression—Depression
CGI-OCD	Clinical Global Impression—Obsessive–Compulsive Disorder
CSSRS	Columbia-Suicide Severity Rating Scale
CSTC	Cortico-Striato-Thalamo-Cortical
CY-BOCS	Children’s Yale–Brown Obsessive–Compulsive Scale
DASS-21	Depression Anxiety Stress Scales (21-item version)
DBS	Deep Brain Stimulation
ECG	Electrocardiogram
GABA	Gamma-Aminobutyric Acid
Glx	Glutamate + Glutamine
HADS-A	Hospital Anxiety and Depression Scale—Anxiety Subscale
HADS-D	Hospital Anxiety and Depression Scale—Depression Subscale
HAM-D/HDRS	Hamilton Depression Rating Scale
H-MRS	Proton Magnetic Resonance Spectroscopy
IDS-SR	Inventory of Depressive Symptomatology—Self Report
IES-R	Impact of Event Scale—Revised
IM	Intramuscular
IP	Intraperitoneal
IV	Intravenous
JBI	Joanna Briggs Institute
KO	Knockout
MADRS	Montgomery–Åsberg Depression Rating Scale
MBT	Marble Burying Test
mGluR5	Metabotropic Glutamate Receptor 5
MK-801	Dizocilpine Maleate
MPFC	Medial Prefrontal Cortex
MRI	Magnetic Resonance Imaging
mTOR	Mechanistic Target of Rapamycin
NBT	Nest Building Test
NMDA	N-Methyl-D-Aspartate Receptor
OCD	Obsessive–Compulsive Disorder
OCD-VAS	Obsessive–Compulsive Disorder Visual Analog Scale
OFT	Open Field Test
OVX	Ovariectomized
PPI	Prepulse Inhibition
PRISMA	Preferred Reporting Items for Systematic Reviews and Meta-Analyses
RCT	Randomized Controlled Trial
RU24969	Serotonin Receptor Agonist with Predominant 5-HT1B Activity
SAPAP3	Synapse-Associated Protein 90/Postsynaptic Density-95-Associated Protein 3
SD	Standard Deviation
SSRI	Selective Serotonin Reuptake Inhibitor
SYRCLE	SYstematic Review Centre for Laboratory animal Experimentation
TR	Treatment Resistant
TRD	Treatment-Resistant Depression
TR-OCD	Treatment-Resistant Obsessive–Compulsive Disorder
vmOFC	Ventromedial Orbitofrontal Cortex
WT	Wild Type
Y-BOCS	Yale–Brown Obsessive–Compulsive Scale
