# Supplementary material for: Ketamine and Esketamine in Obsessive–Compulsive Disorder: A Scoping Review of Clinical and Mechanistic Evidence
Source: Pharmaceuticals (Basel). 2026 Apr 16;19(4):628. doi: 10.3390/ph19040628 (PMC13119091; doi:10.3390/ph19040628)
Supplement: Supplementary file 1 [file pharmaceuticals-19-00628-s001.zip › pharmaceuticals-4239689-supplementary.pdf]

Supplementary Materials

Database Search Strategies

PubMed search query: ("Obsessive-Compulsive Disorder" OR OCD OR obsessive-compulsive OR obsessive compulsive disorder OR obsession OR compulsion) AND (ketamine OR esketamine)

Results retrieved from PubMed: 174

Scopus search query: TITLE-ABS-KEY ("obsessive-compulsive disorder" OR "OCD" OR "obsession" OR "compulsion") AND TITLE-ABS-KEY ("ketamine" OR "esketamine")

Results retrieved from Scopus: 409

Web of Science search query: ALL = (("obsessive-compulsive disorder" OR OCD OR obsession OR compulsion)) AND ALL = ((ketamine OR esketamine))

Results retrieved from Web of Science: 164

Table S1

Preferred Reporting Items for Systematic reviews and Meta-Analyses extension for Scoping Reviews (PRISMA-ScR) Checklist

SECTION	ITEM	PRISMA-ScR CHECKLIST ITEM	REPORTED ON PAGE #
**TITLE**			
Title	1	Identify the report as a scoping review.	1
**ABSTRACT**			
Structured summary	2	Provide a structured summary that includes (as applicable): background, objectives, eligibility criteria, sources of evidence, charting methods, results, and conclusions that relate to the review questions and objectives.	1
**INTRODUCTION**			
Rationale	3	Describe the rationale for the review in the context of what is already known. Explain why the review questions/objectives lend themselves to a scoping review approach.	2-3
Objectives	4	Provide an explicit statement of the questions and objectives being addressed with reference to their key elements (e.g., population or participants, concepts, and context) or other relevant key elements used to conceptualize the review questions and/or objectives.	3
**METHODS**			
Protocol and registration	5	Indicate whether a review protocol exists; state if and where it can be accessed (e.g., a Web address); and if available, provide registration information, including the registration number.	17-18
Eligibility criteria	6	Specify characteristics of the sources of evidence used as eligibility criteria (e.g., years considered, language, and publication status), and provide a rationale.	17-18
Information sources	7	Describe all information sources in the search (e.g., databases with dates of coverage and contact with authors to identify additional sources), as well as the date the most recent search was executed.	17-18
Search	8	Present the full electronic search strategy for at least 1 database, including any limits used, such that it could be repeated.	17
Selection of sources of evidence	9	State the process for selecting sources of evidence (i.e., screening and eligibility) included in the scoping review.	17-18
Data charting process	10	Describe the methods of charting data from the included sources of evidence (e.g., calibrated forms or forms that have been tested by the team before their use, and whether data charting was done	17-18

SECTION	ITEM	PRISMA-ScR CHECKLIST ITEM	REPORTED ON PAGE #
		independently or in duplicate) and any processes for obtaining and confirming data from investigators.	
Data items	11	List and define all variables for which data were sought and any assumptions and simplifications made.	17-18
Critical appraisal of individual sources of evidence	12	If done, provide a rationale for conducting a critical appraisal of included sources of evidence; describe the methods used and how this information was used in any data synthesis (if appropriate).	17-18
Synthesis of results	13	Describe the methods of handling and summarizing the data that were charted.	3-14
**RESULTS**			
Selection of sources of evidence	14	Give numbers of sources of evidence screened, assessed for eligibility, and included in the review, with reasons for exclusions at each stage, ideally using a flow diagram.	3-4,17
Characteristics of sources of evidence	15	For each source of evidence, present characteristics for which data were charted and provide the citations.	17
Critical appraisal within sources of evidence	16	If done, present data on critical appraisal of included sources of evidence (see item 12).	5, 9, 10, 12, 14
Results of individual sources of evidence	17	For each included source of evidence, present the relevant data that were charted that relate to the review questions and objectives.	3-14
Synthesis of results	18	Summarize and/or present the charting results as they relate to the review questions and objectives.	3-14
**DISCUSSION**			
Summary of evidence	19	Summarize the main results (including an overview of concepts, themes, and types of evidence available), link to the review questions and objectives, and consider the relevance to key groups.	14-17
Limitations	20	Discuss the limitations of the scoping review process.	17
Conclusions	21	Provide a general interpretation of the results with respect to the review questions and objectives, as well as potential implications and/or next steps.	18-19
**FUNDING**			
Funding	22	Describe sources of funding for the included sources of evidence, as well as sources of funding for the scoping review. Describe the role of the funders of the scoping review.	19

JBI = Joanna Briggs Institute; PRISMA-ScR = Preferred Reporting Items for Systematic reviews and Meta-Analyses extension for Scoping Reviews.

From: Tricco AC, Lillie E, Zarin W, O'Brien KK, Colquhoun H, Levac D, et al. PRISMA Extension for Scoping Reviews (PRISMAScR): Checklist and Explanation. Ann Intern Med. 2018;169:467–473. doi: 10.7326/M18-0850.

Table S2. SYRCLE Risk of Bias Assessment of Included Preclinical Studies

Article	Study design	Question number									
		1	2	3	4	5	6	7	8	9	10
**Thompson et al., (2020)**	Preclinical experimental animal study	U	U	U	U	U	U	U	Y	U	U
**Gattuso et al.,(2023)**	Preclinical experimental animal study	U	N	U	U	U	U	Y	U	U	U
**Tosta et al.,(2019)**	Preclinical experimental animal study	Y	U	U	U	U	U	U	Y	U	U
**Davis et al.,(2021)**	Preclinical experimental animal study	U	U	U	U	U	U	U	Y	U	U
**Ayub et al.,(2022)**	Preclinical experimental animal study	Y	U	Y	U	Y	U	Y	Y	U	U

1. Was the allocation sequence adequately generated and applied? 2. Were the groups similar at baseline or were they adjusted for confounders in the analysis? 3. Was the allocation adequately concealed? 4. Were the animals randomly housed during the experiment? 5. Were the caregivers and/or investigators blinded from knowledge which intervention each animal received during the experiment? 6. Were animals selected at random for outcome assessment? 7. Was the outcome assessor blinded? 8. Were incomplete outcome data adequately addressed? 9. Are reports of the study free of selective outcome reporting? 10. Was the study apparently free of other problems that could result in high risk of bias?

Table S3. JBI Critical Appraisal of Risk of Bias in Included Randomized Controlled Trials

Article	Study design	Question number												
		1	2	3	4	5	6	7	8	9	10	11	12	13
**Beaglehole et al.,(2024)**	Randomized double-blind crossover trial	Y	U	U	Y	U	Y	Y	Y	Y	Y	U	Y	Y
**Rodriguez et al., (2017)**	Randomized controlled trial (terminated early)	Y	U	U	U	U	Y	Y	Y	Y	Y	U	U	U
**Rodriguez et al., (2015)**	Randomized crossover trial (within-subject design)	Y	U	Y	U	U	Y	Y	Y	Y	U	U	Y	Y
**Rodriguez et al., (2013)**	Randomized double-blind placebo-controlled crossover trial	Y	Y	Y	Y	Y	Y	Y	Y	Y	Y	U	Y	U

1. Was true randomization used for assignment of participants to treatment groups? 2. Was allocation to treatment groups concealed? 3. Were treatment groups similar at the baseline? 4. Were participants blind to treatment assignment? 5. Were those delivering the treatment blind to treatment assignment? 6. Were treatment groups treated identically other than the intervention of interest? 7. Were outcome assessors blind to treatment assignment? 8. Were outcomes measured in the same way for treatment groups? 9. Were outcomes measured in a reliable way? 10. Was follow up complete and if not, were differences between groups in terms of their follow up adequately described and analysed? 11. Were participants analysed in the groups to which they were randomized? 12. Was appropriate statistical analysis used? 13. Was the trial design appropriate and any deviations from the standard RCT design (individual randomization, parallel groups) accounted for in the conduct and analysis of the trial?

Table S4. JBI Critical Appraisal of Risk of Bias in Included Open-Label Studies

Article	Study design	Question number								
		1	2	3	4	5	6	7	8	9
**Bloch et al., (2012)**	Open-label quasi-experimental study	Y	N	N/A	Y	Y	Y	Y	Y	Y
**Beaglehole et al., (2025)**	Open-label quasi-experimental study	Y	N	N/A	Y	Y	Y	Y	U	Y
**Sharma et al., (2020)**	Open-label quasi-experimental study	Y	N	N/A	Y	Y	Y	Y	U	Y

1. Is it clear in the study what is the "cause" and what is the "effect" (i.e. there is no confusion about which variable comes first)? 2. Was there a control group? 3. Were participants included in any comparisons similar? 4. Were the participants included in any comparisons receiving similar treatment/care, other than the exposure or intervention of interest? 5. Were there multiple measurements of the outcome, both pre and post the intervention/exposure? 6. Were the outcomes of participants included in any comparisons measured in the same way? 7. Were outcomes measured in a reliable way? 8. Was follow-up complete and if not, were differences between groups in terms of their follow-up adequately described and analyzed? 9. Was appropriate statistical analysis used?

Table S5. JBI Critical Appraisal of Risk of Bias in Included Case Series Studies

Article	Study design	Question number									
		1	2	3	4	5	6	7	8	9	10
**Kumar et al., (2025)**	Case series (four patients, descriptive)	Y	Y	Y	U	U	Y	Y	Y	Y	N
**Ishimuro et al., (2025)**	Case series (five patients, open-label clinical pilot)	Y	Y	Y	U	Y	Y	Y	Y	Y	N
**Niciu et al., (2013)**	Case series (two-case report)	U	Y	Y	U	U	Y	Y	Y	Y	N

1. Were there clear criteria for inclusion in the case series? 2. Was the condition measured in a standard, reliable way for all participants included in the case series? 3. Were valid methods used for identification of the condition for all participants included in the case series? 4. Did the case series have consecutive inclusion of participants? 5. Did the case series have complete inclusion of participants? 6. Was there clear reporting of the demographics of the participants in the study? 7. Was there clear reporting of clinical information of the participants? 8. Were the outcomes or follow up results of cases clearly reported? 9. Was there clear reporting of the presenting site(s)/clinic(s) demographic information? 10. Was statistical analysis appropriate?

Table S6. JBI Critical Appraisal of Reporting Quality in Included Case Reports

Article	Study design	Question number							
		1	2	3	4	5	6	7	8
**Kaltenboeck et al., (2023)**	Case report	Y	Y	Y	Y	Y	Y	Y	Y
**Rodriguez et al., (2011)**	Case report	Y	Y	Y	Y	Y	Y	Y	Y
**Veraart et al., (2020)**	Case report	Y	Y	Y	N	Y	Y	Y	Y
**Marcatelli et al., (2021)**	Case report	Y	Y	Y	Y	Y	Y	U	Y
**Algin et al., (2024)**	Case report	Y	Y	Y	Y	Y	Y	Y	Y
**Adams et al., (2017)**	Case report	U	Y	Y	Y	Y	Y	Y	Y

1. Were patient's demographic characteristics clearly described? 2. Was the patient's history clearly described and presented as a timeline? 3. Was the current clinical condition of the patient on presentation clearly described? 4. Were diagnostic tests or assessment methods and the results clearly described? 5. Was the intervention(s) or treatment procedure(s) clearly described? 6. Was the post-intervention clinical condition clearly described? 7. Were adverse events (harms) or unanticipated events identified and described? 8. Does the case report provide takeaway lessons?
